# Building an institutional K awardee program at UC Davis through utilization of CTSA resources

**DOI:** 10.1017/cts.2021.839

**Published:** 2021-08-13

**Authors:** Betty P. Guo, Julie Rainwater, Stacey Neves, Erdembileg Anuurad, Ted Wun, Lars Berglund

**Affiliations:** 1 Office of Research, School of Medicine, University of California, Davis, Sacramento, CA, USA; 2 Department of Internal Medicine, University of California, Davis, Sacramento, CA, USA

**Keywords:** K awards, career development, NIH funding, clinical and translational science awards, mentored

## Abstract

NIH offers multiple mentored career development award mechanisms. By building on the UC Davis Clinical and Translational Science Center (CTSC) from its initial NIH funding in 2006, we created an institution-wide K scholar resource. We investigated subsequent NIH funding for K scholars and to what extent CTSC research resources were used. Using NIH RePORTER, we created a database of UC Davis investigators who obtained K01, K08, K23, K25, or K99, as well as institutional KL2 or K12 awards and tracked CTSC research resource use and subsequent funding success. Overall, 94 scholars completed K training between 2007 and 2020, of which 70 participated in one of four institutional, NIH-funded K programs. An additional 103 scholars completed a mentored clinical research training program. Of 94 K awardees, 61 (65%) later achieved NIH funding, with the majority receiving a subsequent individual K award. A higher proportion (73%) of funded scholars used CTSC resources compared to unfunded (48%). Biostatistics and Biomedical Informatics were most commonly used and 55% of scholars used one or more CTSC resource. We conclude that institutional commitment to create a K scholar platform and use of CTSC research resources is associated with high NIH funding rates for early career investigators.

## Introduction

The success of the national scientific enterprise is intrinsically linked to the development of a well-trained cadre of junior investigators [1]. This need is recognized by the National Institutes of Health (NIH), which offers a portfolio of mechanisms to facilitate both the training as well as the transition of junior investigators into research independence [2]. Available funding mechanisms include designated training grants (T), mentored career development awards (K), and special considerations during grant reviews for early stage investigators, i.e., individuals lacking NIH research funding as principal investigators and within 10 years of their terminal degree.

Beyond the NIH, individual universities and academic health centers are important stakeholders in ensuring the successful transition of trainees to independence in a highly competitive environment. Recognizing the challenging nature of this path, many institutions have developed support mechanisms such as protected research time, active mentoring training, peer groups, and assistance with grant writing. Notably, the Clinical and Translational Science Award (CTSA) program, funded by NIH’s National Center for Advancing Translational Sciences, provides institutions with resources to support infrastructure, training, career development, and research and educational resources to catalyze translational research and develop a workforce of biomedical science researchers [3]. While the CTSA program has many goals, its prominent training and education program is a strong indication of NIH’s commitment to training the next generation of scientists [4]. In virtually all CTSA-funded institutions, these resources complement internal institutional efforts to advance trainees and early-career faculty [5,6].

UC Davis received one of the 12 inaugural CTSA awards in 2006 that established the Clinical and Translational Science Center (CTSC) [7,8]. At that time, the institution had few training grants in place and the clinical research curriculum award K30 program, funded only one year earlier, was in its initial phase. The CTSC education programs were designed from the start as platforms to attract funding for its trainees and junior faculty scholars as well as the institution at large by serving as a centralized resource for new training programs. The timely infusion of training resources through the UC Davis CTSC addressed an accumulated need and subsequently empowered the success of both individual and institutional K applicants.

Since its inception, the UC Davis CTSC, including its associated TL1 and KL2 awards, has been continuously NIH funded. With a 15-year history of serving investigators, the CTSC has had the potential to influence the research trajectory of many UC Davis scientists, including early career investigators. In this report, we summarize the impact of the CTSC on the UC Davis training and mentoring environment and the subsequently funded NIH grants received by trainees. Beyond its impact on the number of institutional career development grants awarded, we also sought to determine the role of specific CTSC or affiliated institutional resources on K awardee success rates in obtaining subsequent NIH funding. Although the R01 grant has traditionally been the benchmark for independence, in this age of team science, scientists may also maintain active research programs through funding from different grant mechanisms or as co-investigators or core leaders of another PI’s grant. Thus, in addition to K and R01 funding, we also tracked other “R-type” funding (defined as U, P, or other R funding) as assessment of whether institutional or individual K award funding leads to subsequent NIH funding of any type, providing a relevant metric of K awardee success.

## Methods

### Data set and Measures

We constructed a data set of UC Davis K awardees, both those with individual and institutional K awards, to assess their subsequent funding success in obtaining NIH funding. Using NIH RePORTER, we entered “Davis” in the Organization field, “2007–2020” in the Fiscal Year field, and “Research Career Awards” for the Activity Code. The start year of 2007 was selected as this was the inaugural year for the first scholars entering the CTSA KL2 program. Awardees were included whether they initially received their K award at UC Davis or transferred the award from another institution. In addition, we included scholars who successfully competed for funding through four institutional KL2 or K12 awards (CTSC, the Paul Calabresi NCI K12, the Building Investigator Careers in Women’s Health [BIRCWH] and the Emergency Medicine K12 programs) as well as scholars who entered our K30 (Mentored Clinical Research Training Program [MCRTP]). Attainment of subsequent grant funding was determined from NIH RePORTER and defined as being the Contact PI/Project Leader for any funded grant regardless of grantee organization. R00 awards, which are coupled to K99 awards and contingent upon obtaining a tenure-track equivalent position, were not counted as subsequent funding. Grants in the R, P, or U mechanisms were considered subsequent funding and included irrespective of whether the Contact PI/Project Leader had transferred to another institution. Subsequent funding of R awards was categorized as either R01 or R-type, defined as any non-R01 (including R03 and R21), P or U award.

Scholar use of CTSC resources, defined as scholars having one or more records in the CTSC’s Application for Resource Use (AFRU) on any date after receiving a K award, was captured starting in 2007. Both type of service (Informatics, Biostatistics, Clinical Research Center, Community Engagement and Regulatory Knowledge Support) and frequency of use were captured and included. Grant writing assistance and subsequent leadership positions and honors/awards were also recorded. Leadership positions and honors/awards were self-reported as part of annual scholar surveys.

### Analysis

To correlate subsequent NIH funding with use of UC Davis CTSC resources, we excluded individuals whose K awards were still active in 2020. Individuals were also excluded if they transferred to another institution either during the K award funding period or immediately afterwards, as assessed by online search or NIH RePORTER data of the grantee organization. For the remaining scholars, we determined subsequent funding success rates by calculating, within each category of K award, the percentages of individuals who were awarded any type of NIH research grant following the K award. As these data represent a census that describes the trajectory of funding for K awardees at UC Davis between 2007 and 2020 and are not a sample from a larger population, no inferential statistical tests were conducted.

## Results

### Organizational Impact of UC Davis Clinical and Translational Science Center on Institutional K Programs

At the initiation of the NIH CTSA program, UC Davis had one institutional K program and a few individual K awards. The existing K program, funded a year earlier, was an NIH K30 award with an associated Master’s in Advanced Science degree that provided no salary support and was integrated into the CTSC upon funding of the latter in 2006. As part of the CTSC, the K30 program transitioned into a Mentored Clinical Research Training Program (MCRTP) as one component of the CTSC Research Education and Career Development (RECD) Program. From the beginning of the CTSC program, both the CTSC and School of Medicine leadership realized the opportunity to utilize the center as a platform to create a strong institutional training and mentoring program. This process was facilitated by the K30 Director assuming an overarching role in directing the CTSC RECD Program. By enhancing the resources available through the CTSA with institutional funds and bringing the MCRTP, the CTSC TL1 (pre- and postdoctoral training) and the CTSC KL2 (junior faculty training) programs administratively together, a widely recognized institutional platform and centralized mentoring resource emerged. Another advantage in creating a unified administrative home for the three training programs was the wide recognition by School of Medicine and departmental leadership, that these programs represented a unique opportunity to develop a strong cadre of early-career investigators, ready to take on new leadership roles. Throughout the CTSA funding period, having KL2 or MCRTP scholars as faculty members has been a source of pride and recognition for departments, a sense reinforced by the many successful careers launched through these programs. As detailed in Supplemental Table 1, a number of KL2 and K30/MCRTP graduates have, during the 15-year CTSA funding period, been successful in competing for such leadership positions, many of those at UC Davis.

### K Scholar Community

When the first CTSC KL2 scholar cohort was selected in 2007, several individuals had already graduated from the K30/MCRTP program, providing a source of qualified applicants. Although the CTSC program funded a limited number of K scholars per year due to the relatively small initial size of the NIH award, once established, many features of the program were modeled and leveraged by other subsequent UC Davis institutional K applications. In addition, all UC Davis K scholars, including those not directly funded by the CTSA, were invited to be part of a “K community.” Since inception of the CTSC, 70 K scholars have participated in the four institutional K programs and were joined by 24 scholars receiving individual Ks. In addition, more than 100 scholars have completed MCRTP training (Table 1). As part of this community, all scholars received information about all programs and resources of the CTSC (Supplemental Table 2). Regular meetings for this community included, in addition to “Works-in-Progress” research presentations by the scholars, presentations from directors of the various CTSC programs to promote awareness of CTSC resources and provide easy access for their use. A particularly well-embraced innovation was the establishment of an Annual Scholar Symposium attended by an external Education Advisory Board, which served to strengthen scholar presentation skills and enable valuable extramural mentoring advice. To date, these scholar symposia have been held for 16 years. With scholars from multiple training programs, these joint sessions have resulted in novel collaborations, some of which have led to interdisciplinary research programs. Examples include collaborations of veterinary and medical school trainees, resulting in coclinical trials in human and animal (mostly canine) patients. Other ongoing programs include partnerships between pulmonologists, epidemiologists, and biomedical engineers to analyze breath exhalates to characterize disease severity and exposure, most recently applied to patients exposed to wildfire smoke and to diagnose COVID-19 infections.

**Table 1.** Distribution of gender and underrepresented status of UC Davis MCRTP and K scholars 2007–2020

Training	Number of graduates			
	Total	Females	Males	Under-represented
MCRTP only	103*	56	47	11
CTSC KL2	34	15	19	4
BIRCWH K12	16	13	3	1
Calabresi K12	14	5	9	2
Emergency medicine K12	6	1	5	0
Individual K only	24	13	11	Unknown
Total	197	103	94	18

The table lists scholars who have completed their K or MCRTP training.

Of the 131 MCRTP scholars, 103 did not enter a K program while 28 went on to join four institutional K programs.

The CTSC KL2 Program started in 2007, initially with 2 scholars/year. The BIRCWH K12 Program started in 2006 with a cohort of 5 scholars; one additional scholar started in 2007. The Calabresi K12 Program started in 2012 with a cohort of 2 scholars. The Emergency Medicine K12 Program started in 2012 with a cohort of 2 scholars; when the program ended in 2017, a total of 6 scholars had participated; 4 had completed the program and 2 were current. K scholars that were not part of any of these four institutional programs were designated as individual K scholars.

Data on gender and underrepresented status was collected from institutional K scholars at the time of application. Self-reporting of underrepresented status was voluntary and only 5% of trainees withheld the information.

**Fig. 1.**
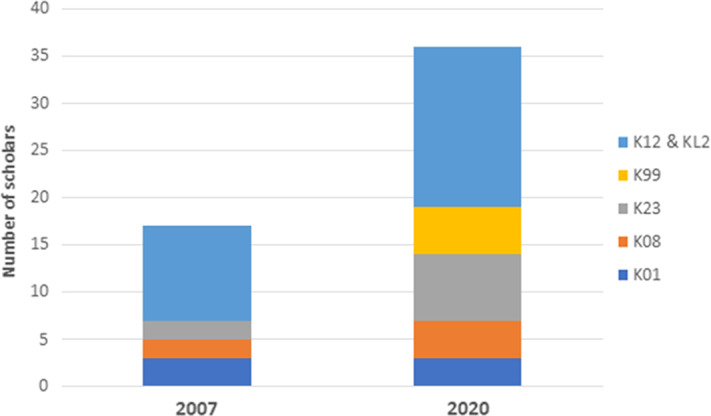
Scholars with funded mentored K awards at UC Davis in 2007 and 2020. For 2020, scholars with active awards were included. Both institutional and individual K awards are shown.

The early institutional embrace of the CTSA RECD program opened the opportunity to build on this foundation for other institutional K12 programs. Over the 15-year period to date, three other K12 programs have been funded, the Paul Calabresi NCI K12 Program, the Building Interdisciplinary Research Careers in Women’s Health (BIRCWH) Program, and the Emergency Medicine Training Program. In addition, the CTSC has brought infrastructure and administrative support to a number of individual mentored K awardees. In 2020, compared to 17 K scholars at the time of the first year of the CTSC KL2 in 2007, there were 36 K scholars at UC Davis (Fig. 1). Overall, 94 NIH-funded K scholars have received mentored training during the 14-year period (since 2007), of which 47 are women and 7 are from underrepresented groups (Table 1).

To further enhance the skill sets of K scholars, an institutionally funded Grants Facilitation (GF) program was created, reporting to the Associate Dean for Research in the School of Medicine (Supplemental Table 2). As the CTSC Director assumed this institutional leadership position a few years after NIH funding, this grant writing resource was functionally closely integrated and streamlined with the CTSC RECD program, albeit remaining fiscally separate. The GF program has, over the years, consistently had three staff members, of which one position was mainly responsible for assistance with career development awards, ensuring strong continuity and familiarity with the scholars and their research areas. They give orientation and information sessions to K and MCRTP scholars and any junior investigator at UC Davis preparing a K or a R submission are encouraged to seek their assistance, frequently by mentors or program leadership. The CTSC director meets weekly with GF program staff and the KL2 directors refer scholars to the GF program when they have generated enough preliminary data to begin developing a grant application. The value of the GF program was recognized such that a similar resource was subsequently created in the NCI-funded UC Davis Comprehensive Cancer Center to facilitate cancer-related grants including career development and early career investigator proposals.

### Funding Success and CTSC Resource Utilization

A critical measure of success for any career development program is the degree to which recipients receive subsequent grant funding. As seen in Table 2, 41 of the 70 scholars participating in the four institutional K programs received subsequent NIH funding, while the number was considerably lower (12 out of 103) for those who only completed the MCRTP. Thus, while the percentage of scholars funded in relation to the total number participating in the MCRTP and institutional K programs was about 30% (12 MCRTP + 41 institutional K scholars out of 173), the percentage of funded institutional K scholars was considerably higher at 59%. The percentage of subsequent grant funding was also high for trainees who received individual K awards (83%).

**Table 2.** Subsequent NIH funding success by CTSC resource utilization

Training	Scholars		Resources used		Resources not used	
	# Funded scholars	# Unfunded scholars	Funded scholars	Unfunded scholars	Funded scholars	Unfunded scholars
MCRTP only	12/103 (12%)	91/103 (88%)	67%	39%	33%	62%
Institutional K:	41/70 (59%)	29/70 (41%)	85%	86%	15%	14%
CTSC KL2	23/34 (68%)	11/34 (32%)	91%	82%	9%	18%
BIRCWH K12	11/16 (69%)	5/16 (31%)	73%	80%	27%	20%
Calabresi K12	5/14 (36%)	9/14 (64%)	80%	89%	20%	11%
Emergency medicine K12	2/6 (33%)	4/6 (67%)	100%	100%	0%	0%
Individual K only*	20/24 (83%)	4/24 (17%)	30%	0%	70%	100%
Total	73/197 (37%)	124/197 (63%)	73%	48%	32%	52%

The table only lists scholars with completed awards and does not include use of grant writing assistance. Institutional K programs: CTSC KL2, BIRCWH K12, Calabresi K12, and Emergency Medicine K12. For institutional K program scholars, other NIH K awards were counted as subsequent funding.

In total, 28 of the K scholars participating in the institutional K programs had previously completed the MCRTP: 18 of 34 (53%) CTSC KL2 scholars, 2 of 16 (13%) BIRCWH K12 scholars, 3 of 14 (21%) Calabresi K12 scholars, and 5 of 6 (83%) Emergency Medicine K12 scholars.

* K01, K08, K23, K25, K99 scholars who were not previously in an institutional K program.

**Fig. 2.**
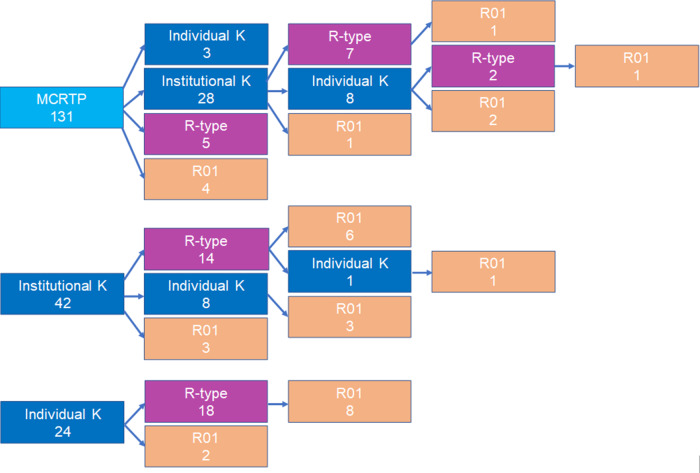
Pathways to NIH funding. Shown are first subsequent NIH grant following institutional or individual K training. R-type funding includes R, U and P grants but not R01. Institutional K programs: CTSC KL2, BIRCWH K12, Calabresi K12, and emergency medicine K12. MCRTP – Institutional K pathway: (7 + 8 + 1)/28 = 16/28 = 57% funded; Institutional K pathway: (14 + 8 + 3)/40 = 25/40 = 60% funded.

Twenty-eight of 70 institutional K scholars initially completed the MCRTP, while 42 scholars were directly recruited into an institutional K program (Fig. 2). The degree of subsequent NIH grant funding was comparable for these two sets of scholars, 57% versus 60% (Fig. 2). Individual K awards were the primary first award obtained after KL2 training, with 16 scholars receiving a K01, K08, or K23 as their first award after completing the KL2 (Fig. 2), while other scholars received an R01 award or an R-type award either individually or as part of a team (as either a multi-PI or leader of a project or core). Notably, the BIRCWH K12, which offers five years of training compared to two to three years for the other programs, had the highest subsequent funding success rate (69%), similar to that of individual K awardees (83%), who also received up to a total of five years of training (Table 2).

We next examined whether utilization of CTSC resources was associated with subsequent grant funding success. As is common for CTSA programs, the UC Davis CTSC offers multiple services to advance translational research, including Informatics, Biostatistics, Clinical Research support, Community Engagement, and Regulatory Knowledge Support (Supplemental Table 2). In addition, activities and services managed by or affiliated with the CTSC are also available, including K scholar cohort activities and grant writing assistance. An institutional initiative to create a UC Davis Health Mentoring Academy, led by CTSC faculty, was launched in 2010. The academy included training for department and center faculty with mentoring responsibilities. It was open to mentees at all stages and every UC Davis K scholar was required to participate. As seen in Table 2, scholars in all institutional K programs had higher success rates for subsequent funding when CTSC resources were utilized.

The CTSC Pilot and Collaborative Studies program was from the inception of the award in 2006 designed to be a resource open to all UC Davis faculty. Specific requirements for funding included a team science focus with co-applicants from different departments, colleges or schools, as well as inclusion of a trainee at any level, from undergraduate or medical students to junior faculty in career development programs. While the number of applications for pilot funding widely exceeded the number of awards, in all 21 of the 94 K scholars received CTSC or Cancer Center pilot funding as PI or co-PI. Nine of these 21 subsequently received NIH funding.

To examine how K and MCRTP scholars used CTSC resources, we extracted data on the distribution of resource use by scholars across the various programs (Informatics, Biostatistics, Community Engagement, etc.) (Supplemental Table 3). Scholars who utilized the CTSC often accessed more than one program, with 109 scholars (55%) using one or more services. Biostatistics and Informatics were most commonly used. Notably, there was similar usage of Grant Writing Assistance.

## Discussion

Ensuring a successful career trajectory for research faculty is an important institutional priority. As NIH inflation-adjusted funding paylines have remained flat and team science is steadily growing in importance, the need to develop an accessible and well-resourced research infrastructure at the institutional level has become more widely recognized [4,9–12]. The CTSA programs are ideal vehicles to house and oversee many specialized functions, such as informatics, biostatistics, clinical trials administration and community engagement [7]. Research training, education, and career development are institutional priorities but, traditionally, T and K training programs have been disciplinary and more rarely integrated in an interdisciplinary and interprofessional fashion. The CTSA mechanism offers unique advantages in this regard, building bridges to other disciplines such as nursing, engineering, environmental and social sciences, and creating research opportunities for novel teams [13,14].

In the present paper, we describe our 15-year history of utilizing the CTSA training programs as a foundation upon which to attract and build additional institutional training programs. At UC Davis, the CTSC served in a trailblazing function, breaking new ground that paved the way to develop other training programs and generate extramural funding. Many of the resources inherent in a CTSA program provide wide benefit for trainees in other training programs, regardless of discipline. In addition, the interdisciplinary focus of the CTSA program makes it ideal to serve as an “honest broker,” generating synergies across disciplines. Notably, this CTSA role has made the CTSC a critical pillar in UC Davis’ plans to create an innovation hub, Aggie Square, that also centers on lifelong learning [15]. Once other K programs were obtained, a community of K scholars was developed, leading to cross-fertilization of ideas and approaches as well as new collaborations. Being part of a K community also promotes peer mentoring, an important component of career growth and team building [16]. This sense of community was facilitated by a shared administration that continued to bring the different training programs together [4].

Due to the existence of a K scholar community, we were able to examine the effect of a CTSA on NIH grant funding for individuals who had received a mentored K. Because mentored Ks are early career stage awards, focusing on this group enabled us to assess the impact of a CTSA on trainees without substantial previous funding. We included K01, K08, K23, K25, and K99 awardees, representing the majority of NIH extramural mentored career development award mechanisms. These data show that the combination of intensive research training and individual grant writing assistance has a substantial impact on successfully transitioning KL2 scholars to individual NIH mentored career development awards, thus positioning them for success. This difference was particularly noteworthy when comparing K scholars to MCRTP scholars. Nationally, NIH individual K awardees have a 24% higher likelihood of receiving subsequent R01 or equivalent research project grant funding [17–21]. The large relative increase in K08 and K23 awards also suggested that the program positively impacted the career trajectory for physician scientists at the institution.

Some CTSC resources were more heavily used than others, likely due to the nature of scholar research topics or access to similar resources elsewhere, either through their department, mentors or collaborators. Projects analyzing health records or large data sets have become increasingly common, reflected in Informatics and Biostatistics being the two CTSC resources used by the most scholars. In addition, these resources have applicability across a range of research topics. Although most scholars are exposed to Informatics and Biostatistics through coursework, the heavy use of these resources indicates that some projects may benefit from specialized expertise.

As part of integrating the CTSC with the Grants Facilitation (GF) program, scholars are referred to the GF program well in advance of training completion to allow adequate time to plan, strategize, and review proposal drafts. In addition, the GF program makes scholars aware of special funding opportunities (such as those for early stage investigators and the recent Small Grant Program [R03] for the NCATS CTSA Program). By guiding scholars through every step of what is typically their first NIH application, the resource serves as an additional layer of hands-on mentorship in grant writing and submission. The existence of a K community provides a means to collectively learn from both successful and unsuccessful applications. Use of CTSA and affiliated institutional resources, such as grant writing assistance, was more common among scholars who received subsequent NIH funding, with the exception of individual K scholars.

An important factor to consider is length of the mentored training period. Among the four institutional K programs at UC Davis, the BIRCWH program has offered a full 5-year funding period, while the other programs have limited the funding period to 2 or 3 years. In some cases, the third year was institutionally funded. It is therefore not surprising that the success rate for subsequent grant funding following the institutional K award was highest for BIRCWH scholars. The average time on grant for BIRCWH scholars was ∼3.5 years, with nearly half (5 PhDs/DrPHs, 1 MD and 1 MD, PhD) exiting the program before 5 years. Nevertheless, subsequent funding success was comparable between those who exited early and those who did not. Although not all scholars needed the 5 years, those who required additional time to generate preliminary data or resubmit grant applications were able to maintain continuity in their research. This is also likely contributing to the higher success rate of individual K scholars, who also received 5 years of funding [17]. These two cohorts are also comparable in that they transition to R01 or R-type funding, as their length or type of K excludes them in most cases from further NIH K awards. This finding suggests the importance of supplementing more time-limited institutional K awards with time extensions and with individual K awards as the time it takes for investigators to obtain their first R01 or R01-equivalent funding has become increasingly longer [1].

As biomedical research, particularly in clinical and translational science, is becoming more complex and increasingly requires a team science approach, it is relevant for trainees to consider opportunities to contribute as part of a larger grant team. Although R01 funding largely remains a standard measure of research independence and success, an equally important measure may be a role as collaborators, multi-PI or project or core leaders of P or U grants. In addition, smaller research project awards such as R03 or R21 grants represent important mechanisms for maintaining research productivity. It was notable that such types of non-R01 research grant funding were more common as the next step for both institutional and individual K awardees than R01 funding. This is likely to become even more commonplace in the future. Several of these larger P or U grants represented long-running, continuously funded grants. Given the firm institutional anchoring of K scholars in the four institutional K programs, these scholars provided a well-trained cadre for succession in leadership of large, established research programs.

We acknowledge some limitations of our study. As our focus was primarily on NIH funding, we did not take funding from other agencies into account. We also did not consider payline fluctuations over time, differences across institutes, or that paylines for K and R awards may differ.

In conclusion, our results demonstrate a trajectory of growth for mentored career development awards at our institution. UC Davis rapidly recognized the importance of a CTSA award and used this as a springboard to build a training platform that has generated a cadre of NIH-funded investigators as well as individuals who have assumed various leadership positions. These data further indicate an advantage for scholars who used CTSC services and provide important insight into the effect of a CTSA on scholar career trajectories. The competitiveness of NIH grants makes starting with a K award as a springboard a good strategy for obtaining any type of subsequent NIH funding. While an R01 award is an indicator of research independence, it is not the only benchmark of success or continued contribution in research. Our data also point to the importance of having a strategic institutional plan to ensure that trainees participating in institutional K programs receive the full training period in order to optimize their career trajectories. Our experience to date has informed institutional leadership, program leaders, and not least future trainees positioning UC Davis in a strong position to continue to build on this foundation.

## Supplementary material

For supplementary material accompanying this paper visit http://dx.doi.org/10.1017/cts.2021.839. click here to view supplementary material
