# Supplementary material for: Building an institutional K awardee program at UC Davis through utilization of CTSA resources
Source: J Clin Transl Sci. 2021 Aug 13;5(1):e171. doi: 10.1017/cts.2021.839 (PMC8532183; doi:10.1017/cts.2021.839)
Supplement: Supplementary file 1 [file S2059866121008396sup001.docx]

| **Supplemental Table 1. Examples of Leadership Roles and Awards Attained by Graduates of UC Davis CTSC KL2 and MCRTP Programs** | | |
| --- | --- | --- |
|  | **Institution/**  **Program** | **Leadership Role** |
| **KL2 Graduates** | **UC Davis** | - Assistant Dean for Research - Division Chief - 3 Program Directors |
|  | **National, State or Other Academic Institutions** | - Associate Dean for Research - Branch Chief NIH - Deputy Associate Chief of Staff, VA - Clinical Quality Officer, California Department of Health Care Services - Chief Medical Office, Research Institute - Member, Governor’s Task Force - Associate Vice President, National Federation - Distinguished Clinical Research Achievement Award, Clinical Research Forum |
|  | **CTSA Program** | - Program Leaders in 2 CTSA Centers |
| **MCRTP Graduates** | **UC Davis** | - PI, NIH Training Program - 3 Division Chiefs - 2 Program Directors - Chair, Graduate Group |
|  | **CTSA Program** | - CTSA Associate Program Director - CTSA Program Director |
|  | **Institution** | **Awards** |
| **KL2 and MCRTP Graduates** | **UC Davis or Other Academic Institution** | - 9 Dean’s Awards (Mentoring, Team Science, Research Excellence) - 5 Departmental Research Awards - 10 National Foundation or Society Research Awards - Sautter Award for Information Technology |
| VA = Veterans Affairs; NIH = National Institutes of Health; CTSA = Clinical and Translational Science Award  Graduate groups are interdisciplinary research groups to which graduate students belong at UC Davis. | | |

| **Supplemental Table 2. Resources Available to Scholars** | |
| --- | --- |
| **CTSC Resources** | |
| **Resource** | **Services** |
| Informatics | - Access to clinical and translational informatics tools, data resources, training - Support and expand capabilities for securely designing and analyzing clinical and integrative data-driven research |
| Biostatistics | - Develop protocols, statistical plans, data safety monitoring plans, data analysis - Contribute to the statistical sections of grant applications, abstracts, and manuscripts |
| Clinical Research Center | - Access to unique equipment and protocol administration tools - Staff trained in human subjects’ protection, good clinical practices, protocol implementation, and compliance |
| Community Engagement | - Link investigators and community partners - Help researchers forge relationships with community partners, California health agencies, and elected officials |
| Regulatory Knowledge Support | - Education in regulatory and clinical research administration, assist with study recruitment, guide investigational teams through local, state, and federal requirements - Support for IRB and FDA applications, research budgeting and billing, and cost analysis - Access to clinical research coordinators who can help monitor quality assurance through all phases of a clinical research protocol - Assistance with clinicaltrials.gov |
| **Resources Managed by or Affiliated with the CTSC** | |
| **Resource** | **Activities/Services** |
| Education and Career Development | - Works-in-Progress seminars - Annual scholar symposium - K scholar community |
| Grant Writing Assistance | - Identification of funding opportunities and navigation of funding agency policies - Editing and proposal development - Summary statement review and strategy for next steps |
| IRB = Institutional Review Board; FDA = Federal Drug Administration.  Grant writing assistance was available through Grants programs affiliated with the CTSC and the Comprehensive Cancer Center. | |

| **Supplemental Table 3. Resource Use** | | |
| --- | --- | --- |
| **CTSC Resource** | **# Used*** | **%** |
| Informatics | 67 | 34% |
| Biostatistics | 72 | 37% |
| Clinical Research Center | 40 | 20% |
| Community Engagement | 5 | 2% |
| Regulatory Knowledge Support | 32 | 16% |
| **CTSC-affiliated Resource** | **# Used*** | **%** |
| Grant Writing Assistance | ~71 | N/A |
| CTSC = Clinical and Translational Science Center; N/A = not applicable.  * Number of scholars who requested indicated resource out of 197 scholars. Individual scholars typically requested use of more than one resource. 109/197=55% of scholars used CTSC resources.  Grant writing assistance was available through Grants programs affiliated with the CTSC and the Comprehensive Cancer Center. Use of this resource is not captured electronically, therefore the number of scholars who used this service is approximate and a percentage is not shown. | | |
